# IMPACT OF TGF-β FAMILY-RELATED GROWTH FACTORS ON CHONDROGENIC DIFFERENTIATION OF ADIPOSE-DERIVED STEM CELLS ISOLATED FROM LIPOASPIRATES AND INFRAPATELLAR FAT PADS OF OSTEOARTHRITIC PATIENTS

**DOI:** 10.22203/eCM.v035a15

**Published:** 2018-04-13

**Authors:** E. López-Ruiz, G. Jiménez, W. Kwiatkowski, E. Montañez, F. Arrebola, E. Carrillo, S. Choe, J.A. Marchal, M. Perán

**Affiliations:** 1 Department of Health Sciences, University of Jaén, Jaén, Spain; 2 Biopathology and Regenerative Medicine Institute (IBIMER), Centre for Biomedical Research, University of Granada, Granada, Spain; 3 Department of Human Anatomy and Embryology, Faculty of Medicine and Excellence Research Unit “Modelling Nature” (MNat), University of Granada, Spain; 4 Biosanitary Research Institute of Granada (ibs.GRANADA), University Hospitals of Granada-University of Granada, Granada, Spain; 5 Drug Discovery Collaboratory, Qualcomm Institute, University of California, La Jolla, California, USA; 6 Department of Orthopaedic Surgery and Traumatology, Virgen de la Victoria University Hospital, Málaga, Spain; 7 Institute of Biomedical Research in Malaga (IBIMA), Málaga, Spain; 8 Department of Histology, Faculty of Medicine, University of Granada, Granada, Spain

**Keywords:** Adipose stem cells, chondrogenic differentiation, osteoarthritis, transforming growth factor-β family-related growth factors

## Abstract

The success of cell-based approaches for the treatment of cartilage defects requires an optimal autologous cell source with chondrogenic differentiation ability that maintains its differentiated properties and stability following implantation. The objective of this study was to compare the chondrogenic capacity of mesenchymal stem cells (MSCs) isolated from lipoaspirates (ASCs) and infrapatellar fat pads (IFPSCs) of osteoarthritic patients and treated with transforming growth factor (TGF)-β family-related growth factors. Cells were cultured for 6 weeks in a 3D pellet culture system with the chimeric activin A/bone morphogenic protein (BMP)-2 ligand (AB235), the chimeric nodal/BMP-2 ligand (NB260) or BMP-2. To investigate the stability of the new cartilage, ASCs-treated pellets were transplanted subcutaneously into severe combined immunodeficiency (SCID) mice. Histological and immunohistochemical assessment confirmed that the growth factors induced cartilage differentiation in both isolated cell types. However, reverse transcription-quantitative PCR results showed that ASCs presented a higher chondrogenic potential than IFPSCs. *In vivo* results revealed that AB235-treated ASCs pellets were larger in size and could form stable cartilage-like tissue as compared to NB260-treated pellets, while BMP-2-treated pellets underwent calcification. The chondrogenic induction of ASCs by AB235 treatment was mediated by SMAD2/3 activation, as proved by immunofluorescence analysis. The results of this study indicated that the combination of ASCs and AB235 might lead to a cell-based cartilage regeneration treatment.

## Introduction

Cartilage lesions are common and often lead to further cartilage loss and development of osteoarthritis (OA). Articular cartilage has a very low self-repair potential due to its avascular nature and low cellular mitotic activity. Therefore, cell-based therapies directed to repair chondral lesions have emerged as a promising new biological approach. Autologous chondrocyte implantation (ACI) (Brittberg *et al*., 1994) is currently used in clinic. However, the use of ACI is limited to small injuries, since only a small number of chondrocytes can be isolated from a patient biopsy. To increase the number of freshly isolated chondrocytes, the cells must be expanded *in vitro*, but prolonged monolayer culture induces dedifferentiation of the chondrocyte (Ma *et al*., 2013). In this regard, adult mesenchymal stem cells (MSCs), which can be isolated, cultured and differentiated into a range of specialised cell types, are proposed as an alternative cell source for treating cartilage damage (Oldershaw, 2012). Differentiation potential varies widely among MSCs derived from different tissues; consequently, the identification of the optimal cell source is a pre-requisite for their clinical use. Adipose tissue represents an ideal choice due to its easy accessibility and abundance in the body.

Adipose derived stem cells (ASCs) are commonly isolated from the liposuction aspirate, although other sources, such as the infrapatellar fat pad (IFP), are described (Buckley *et al*., 2010; López-Ruiz *et al*., 2013). Stem cells isolated from the IFP (IFPSCs) adhere and undergo chondrogenesis in monolayer and 3D culture (López-Ruiz *et al*., 2013; Liu *et al*., 2014). These properties, together with their orthotopic localisation and easy accessibility, make them a potential cell source for the treatment of cartilage defects in the knee.

Several members of the TGF-β superfamily induce chondrogenic differentiation of MSCs, in particular bone morphogenetic protein 2 (BMP-2) (Liao *et al*., 2014; Zhou *et al*., 2016). BMP-2 can also induce osteogenic differentiation and promote endochondral ossification (Zhou *et al*., 2016; Vanhatupa *et al*., 2015). Consequently, optimising BMP-2 and potentiating its specific chondrogenic ability is a challenge and a key step in the process of cartilage formation. Previous work established a library of TGF-β chimeric ligands, using a segment-swapping strategy to combine BMP-2 and activin A or nodal sequences (Perán *et al*., 2013; Esquivies *et al*., 2014). From these libraries, two chimeric ligands, activin A/BMP-2 (AB235) and nodal/BMP-2 (NB260), with powerful chondrogenic induction potential, were identified (Perán *et al*., 2013; Esquivies *et al*., 2014; Jiménez *et al*., 2015).

The aim of the present study was to present an *in vitro* model for cartilage cell therapy. The chondrogenic potential of MSCs isolated from liposuctions (ASCs) and infrapatellar fat pads of OA patients (IFPSCs) was compared. In addition, three different chondrogenic induction factors, AB235, NB260 and BMP-2, were evaluated. The study compared 6 different protocol strategies to establish the best combination of stem cells and chondrogenic factor for cell therapy applications.

## Materials and Methods

### Patients

Human IFPSCs were obtained from patients with knee OA (*n* = 8) during joint replacement surgery. The clinical and demographics features of the OA patients are listed in Table 1. None of the patients had a history of inflammatory arthritis or crystal-induced arthritis. Infrapatellar (Hoffa’s) fat pads were harvested from the interior of the capsules, excluding vascular areas and synovial regions. Samples collected during joint arthroplasty were transported to the laboratory in Dulbecco’s modified Eagle’s medium (DMEM; Sigma-Aldrich) with 100 U/mL penicillin and 100 mg/mL streptomycin. Human abdominal fat was obtained from healthy donors (*n* = 8) undergoing liposuction plastic surgery (range of age 44-61). All samples used in this study were collected with informed consent and Institutional Review Board approval (ethic permission number: 02/022010 Hospital Virgen de la Victoria, Málaga, Spain).

### Isolation and culture of MSCs

MSCs were isolated from lipoaspirates’ adipose tissue or from IFP tissue. Briefly, lipoaspirates were washed and treated for 1 h at 37 °C with 1 % collagenase type IA (Sigma-Aldrich) in Hank’s Balanced Salt Solution (Sigma-Aldrich) under gentle agitation. The digested tissue was centrifuged and the supernatant eliminated. The cell pellet obtained was washed twice in DMEM supplemented with 10 % foetal bovine serum (FBS) and re-suspended in growth medium. Fat tissue from IFP was finely minced, digested using enzymatic solution of 1 % collagenase type IA (Sigma-Aldrich) and incubated on a shaker at 37 °C for 1 h. After digestion, collagenase was removed by a single wash in sterile phosphate-buffered saline (PBS), followed by two further washes in DMEM supplemented with 10 % FBS. Approximately 1.8 × 10^5^ (range 1.6-2 × 10^5^) cells from 1 g of lipoaspirate and 1.1 × 10^5^ (range 9-1.3 × 10^5^) from 1 g of IFP were obtained. Cells from both sources were resuspended in DMEM (Sigma-Aldrich) containing 10 % FBS and 1 % penicillin/streptomycin and seeded at equal densities (5 × 10^3^ cells/cm^2^) into T-75 culture flasks with 10 mL of culture medium and cultured in an incubator at 37 °C, 21 % O_2_ and 5 % CO_2_. The medium was changed every 3 d. Freshly isolated cells were grown in monolayer culture up to passage 4-5 (P4-5) at a seeding density of 5 × 10^3^ cells/cm^2^ at each passage.

Phenotype and differentiation potential of isolated cells were characterised, as previously described (López-Ruiz *et al*., 2013; Perán *et al*., 2010). To examine their immunophenotype, cells were trypsinised, washed and resuspended in PBS with 1 % bovine serum albumin (BSA; Sigma-Aldrich). A total of 2 × 10^5^ cells was incubated in the dark for 30 min with fluorochrome-conjugated monoclonal antibodies [CD34, CD45, CD90, CD73, CD105 and CD133 (Miltenyi Biotec, Auburn, CA, USA)], washed in PBS and analysed by flow cytometry in a FACSCanto II cytometer (BD Biosciences). For the differentiation assays, ASCs and IFPSCs were plated at 1 × 10^5^ cells/cm^2^ in DMEM-FBS into 6-well culture plates. After 48 h, the culture medium was replaced with specific differentiation-inductive medium. For adipogenic, osteogenic and chondrogenic differentiation, cells were cultured for 2 weeks in hMSC Adipogenic Differentiation BulletKit™ (Lonza), hMSC Osteogenic Differentiation BulletKit™ (Lonza) and StemMACS ChondroDiff Medium (Miltenyi Biotec), respectively. Differentiated cell cultures were stained with oil red O (Amresco, Solon, OH, USA) for adipogenic differentiation, alizarin red (Lonza) for osteogenic differentiation or toluidine blue (Sigma-Aldrich) for chondrogenic differentiation.

### Proliferation assay

Cellular proliferation was tested using the sulphorhodamine B (SRB) assay. ASCs and IFPSCs from 2 donors were plated at 1 × 10^4^ cells/well in triplicate into 24-well plates and cultured in a humidified atmosphere at 37 °C and 5 % CO_2_ for 7 d. Cells were fixed for 1 h at 4 °C on day 1, 3, 5 and 7 by the addition of 125 μL of 10 % ice-cold trichloroacetic acid. After fixation, medium was removed, cells were washed and total biomass was determined by SRB assay (Sigma-Aldrich) (250 μL of 0.4 % SRB; 30 min). Unincorporated dye was removed by washing with 1 % acetic acid, whilst cell-incorporated dye was solubilised using 10 mM Tris-base. Dye incorporation, reflecting cell biomass, was measured at 492 nm, using a microplate reader (MB-580-HEALES, Shenzhen, China).

### Chondrogenic differentiation in cell pellet culture

Cells were grown on pellet culture following a modification of the protocol described by Estes *et al*. (2010). Briefly, 1 × 10^5^ cells were grown in monolayer in a 6-well plate for 2 weeks, then the monolayer was manually separated using a sterile tip, carefully transferred to a 15 mL conical tube and incubated for 4 weeks with loosened tops at 37 °C and 5 % CO_2_. The medium was changed every other day for the duration of the experiment and the tubes were gently shaken to avoid the adherence of the pellet to the plastic walls. Control cells were grown in high glucose DMEM (Sigma-Aldrich), supplemented with 10 % FBS (Sigma-Aldrich), 50 μg/μL of L-ascorbic acid 2-phosphate (Sigma-Aldrich), 1 % penicillin-streptomycin (Sigma-Aldrich) and 1 % insulin-transferrin-selenium (ITS; Gibco). To direct chondrogenic differentiation, 10 ng/mL of the chondrogenesis-inducing factors (NB260, AB235 or BMP-2) were added fresh during each medium change every 48 h. NB260 and AB235 chimeric ligands and BMP-2 were generated as previously described (Allendorph *et al*., 2011).

### Histological and immunohistochemical analysis

Cartilage tissue and cell pellets were immersed in 4 % paraformaldehyde in 0.1 M PBS for 4 h at 4 °C, washed in 0.1 M PBS and embedded in paraffin in an automatic tissue processor (TP1020, Leica). The paraffin blocks were cut into 4 μm-thick sections for staining. Alcian blue and toluidine blue revealed the presence of glycosaminoglycans (blue and purple, respectively), Masson’s trichrome of collagens (green) and alizarin red of calcified matrix (red).

For immunofluorescence, pellets were fixed with 4 % paraformaldehyde in PBS for 20 min at room temperature (RT) and placed in 30 % sucrose over 6 h at 4 °C, to allow tissue to sink. Pellets samples were embedded in optimal cutting temperature (OTC) compound. Blocks were stored at −80 °C, sectioned on a cryostat (CM1510S; Leica) into 8 μm-thick sections and placed on Polysine™ slides (Fisher Scientific). For intracellular staining, sections were permeabilised with 0.1 % Triton X-100 for 15 min, blocked for 1 h at room temperature with 5 % BSA, 5 % FBS in PBS and incubated with the primary antibody overnight at 4 °C. Primary antibodies used were: ColI (SC25974, Santa Cruz Biotechnology), ColII (SC52658, Santa Cruz Biotechnology), Sox 9 (AB5535, EMD Millipore) or p-Smad2/3 (SC11769, Santa Cruz Biotechnology). On the next day, samples were washed thrice with PBS, incubated with the secondary antibodies (Alexa) for 1 h at RT, washed thrice with PBS and mounted with 4′,6-diamidino-2-phenylindole (DAPI)-containing mounting medium. Images were acquired with a Leica DM 5500B microscope.

### Quantitative image analysis

Quantitative image analysis was performed using ImageJ™ software (v1. 43, NIH), as previously described (Jensen, 2013; McCloy *et al*., 2014).

### RNA isolation and reverse transcription-quantitative PCR (RT-qPCR) analysis

Total cellular RNA was isolated using TriReagent (Sigma-Aldrich) and reverse transcribed using the Reverse Transcription System kit (Promega). RT-qPCR was performed using the SYBR-Green PCR Master mix (Promega) according to the manufacturer’s recommendations. PCR reactions were performed as follows: an initial denaturation step at 95 °C for 2 min, 40 cycles at 95 °C for 5 s and 60 °C for 30 s and a final dissociation cycle at 95 °C. The gene expression levels were normalised to corresponding *GAPDH* values and are shown as fold change relative to the control sample. All the samples were analysed in triplicate for each gene. Primer sequences used are listed in Table 2.

### *In vivo* assay

**Fig. 1:**
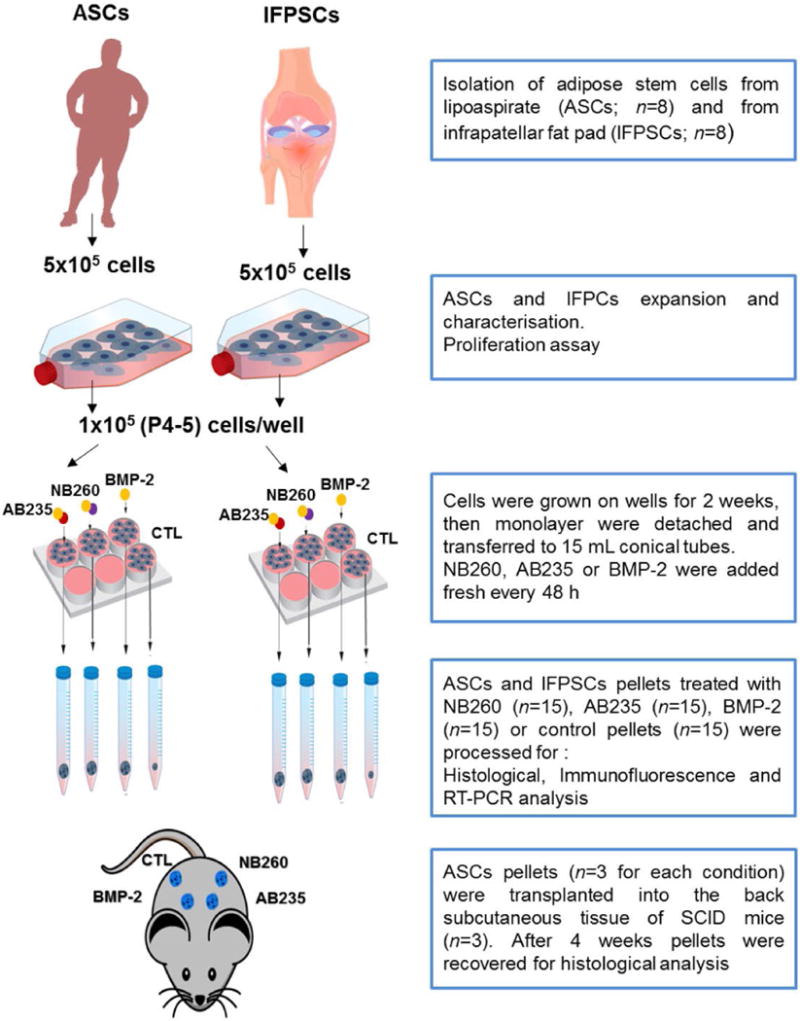
Flow chart of the study showing the experimental design.

After 6 weeks of *in vitro* chondrogenic induction, NB260-, AB235- or BMP-2-treated and control ASCs pellets (3 pellets for each condition) were transplanted into subcutaneous pockets of 3 severe combined immunodeficiency (SCID) mice, thus each mouse received 4 pellets (Fig. 1). The procedure is described in Pelttari *et al*. (2006) and Liu *et al*. (2008). Briefly, before surgery, mice were anaesthetised by isoflurane inhalation, the skin on both lateral sites of the spine was cleaned with 70 % alcohol and 4 subcutaneous pockets were created in each mouse. The pellets were inserted, attached to the wall of the pocket by a small amount of fibrin glue, and the pockets closed. Mice were maintained under standard conditions and provided with food and water *ad libitum*. After 4 weeks, the mice were sacrificed by an overdose injection of anaesthetic and the pellets were excised for further histologic analysis. *In vivo* assays were carried out in accordance with the approved guidelines of the University of Granada, Spain following institutional and international standards for animal welfare and experimental procedure. All experimental protocols were approved by the Research Ethics Committee of the University of Granada, Spain.

### Statistical analysis

Significant differences between treatments were tested using one-way ANOVA and Fisher least significant difference (LSD) test. Assumptions of analysis of variance were tested and confirmed by using transformed data sets [log (dependent variable value + 1)], when necessary. All the data are presented as mean ± standard deviation of 3 independent experiments and deemed statistically significant for *p* < 0.01.

## Results

### Evaluation of the chondrogenic differentiation potential of TGF-β family-related growth factors in ASCs and IFPSCs

**Fig. 2:**
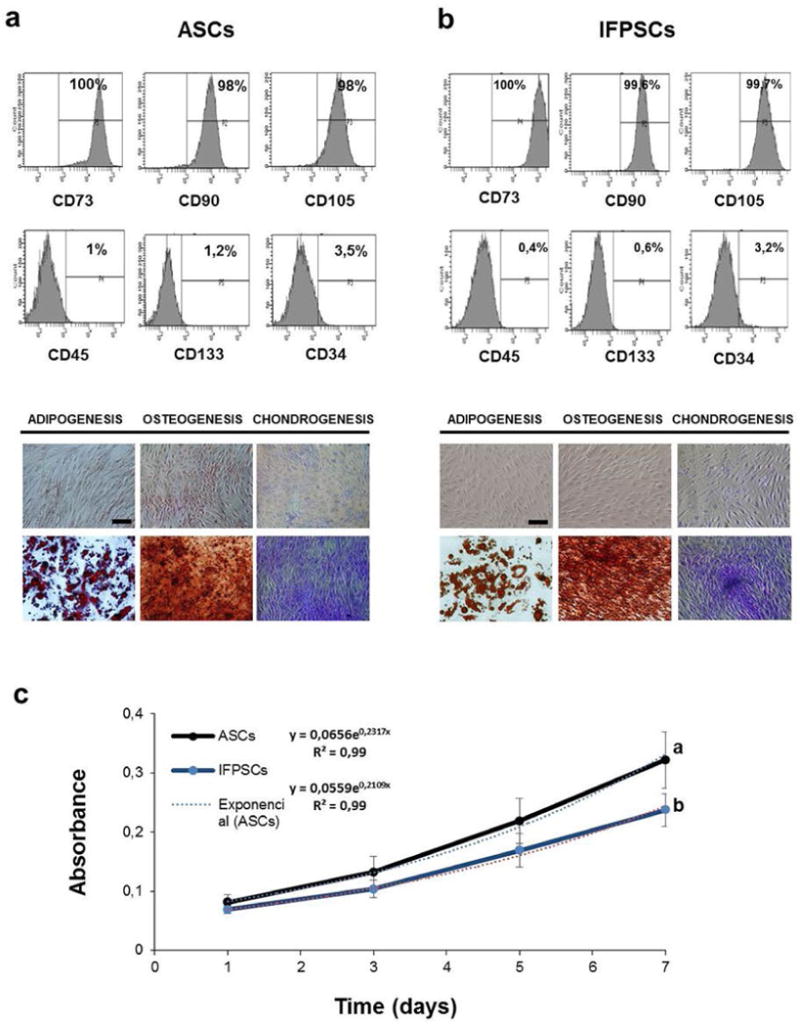
Phenotypic characterisation and differentiation potential of (**a**) ASCs and (**b**) IFPSCs. (**a**) FACS characterisation of ASCs showed a positive expression of the surface markers CD73 (100 %), CD90 (98 %), CD105 (98 %) and a negative expression of CD45 (1 %), CD133 (1.2 %) and CD34 (3.5 %). (**b**) FACS characterisation of IFPSCs showed a positive expression of the surface markers CD73 (100 %), CD90 (100 %), CD105 (100 %) and a negative expression of CD45 (0.4 %), CD133 (0.6 %) and CD34 (3.2 %). The differentiation potential of ASCs and IFPSCs towards adipogenic, osteogenic and chondrogenic lineage was confirmed by oil red O, alizarin red S and toluidine blue staining, respectively. (**a,b**) Upper pictutes show negative controls, cells cultured in normal medium for 2 weeks and then histochemically stained. Scale bars: 200 μm. (**c**) Cellular proliferation of ASCs and IFPs tested by the SRB assay. Absorbance at 492 nm, as proxy of cell biomass. Exponential function fit and regression coefficient are shown. Different letters stand for significant differences in the absorbance (one-way ANOVA, *p* < 0.05).

Isolated ASCs and IFPSCs were characterised, following the established criteria of the International Society for Cellular Therapy (ISCT), to define multipotent mesenchymal stromal cells; cells that were plastic-adherent, expressed specific surface antigens and had multipotent differentiation potential (Fig. 2a,b). Proliferation assay showed that ASC and IFPSCs had similar doubling times, with a slightly higher value for ASCs when compared with IFPSCs (3.9 and 4.2 d, respectively), although not statistically significant (Fig. 2c).

**Fig. 3:**
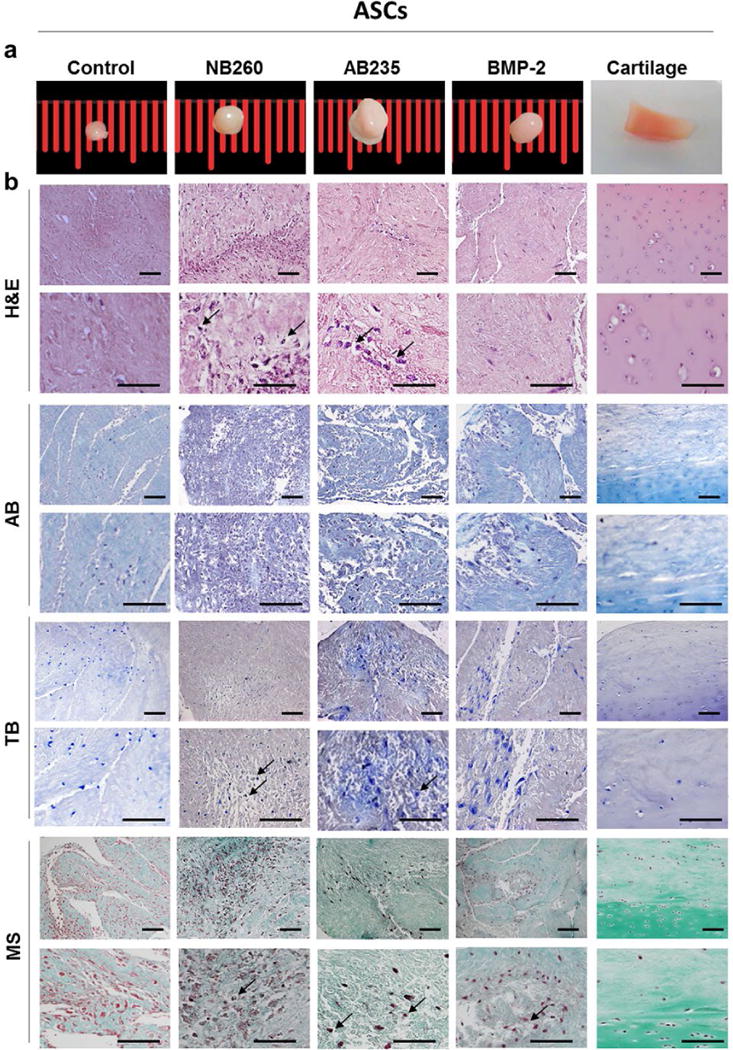
Chondrogenic induction of ASCs with TGF-β family-related growth factors. (**a**) Representative pictures of ASCs cultured in chondrogenic medium for 6 weeks, treated with 10 ng/mL of NB260, AB235 or BMP-2. Untreated pellets and native cartilage tissue were used as control. (**b**) Histological staining of pellets sections with haematoxylin-eosin (H&E), alcian blue (AB), toluidine blue (TB) and Masson’s Trichrome (MS) showed the typical morphology of the native cartilage and the presence of collagens and GAGs in the ECM. Scale bars: 100 μm.

**Fig. 4:**
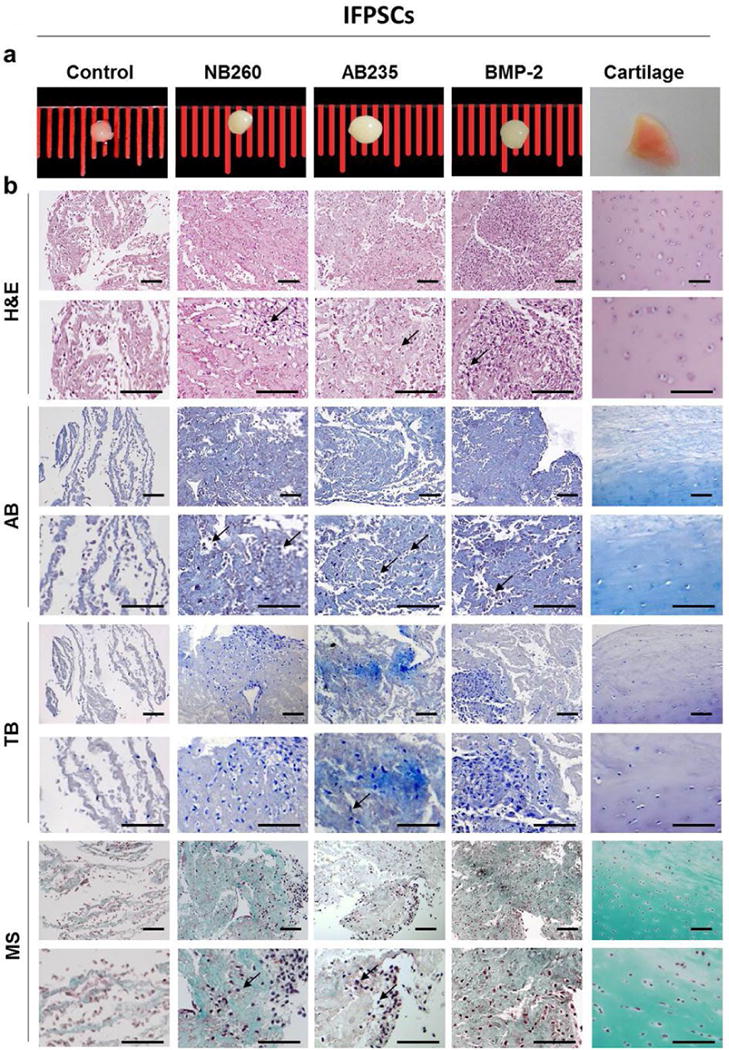
Chondrogenic induction of IFPSCs with with TGF-β family-related growth factors. (**a**) Representative pictutes of IFPSCs cultured in chondrogenic medium for 6 weeks, treated with 10 ng/mL of NB60, AB235 or BMP-2. Untreated pellets and native cartilage tissue were used as control. (**b**) Histological staining of pellets sections with haematoxylin-eosin (H&E), alcian blue (AB), toluidine blue (TB) and Masson’s trichrome (MS) showed the typical morphology of the native cartilage and the presence of collagens and GAGs in the ECM. Scale bars: 100 μm.

ASCs and IFPSCs were cultured in a pellet system and induced to differentiate toward a chondrogenic linage by the addition to the medium of NB260, AB235 or BMP-2. 15 cell pellets for each condition, or without media supplementation (control), were processed for histology, immunofluorescence or RT-qPCR analysis (Fig. 1). ASCs and IFPSCs pellets treated with any of the chondrogenic factors (NB260, AB235 or BPM-2) displayed a larger size (with ASCs being larger than IFPSCs) with a shiny and compact appearance, similar to native cartilage, when compared to untreated control, showing a similar size and less consistent appearance (Fig. 3a and Fig. 4a). Interestingly, ASCs and IFPSCs pellets cultured in AB235-containing medium were larger in comparison to NB260- and BMP-2-induced pellets.

Histological analysis of pellet sections revealed a marked difference between non-treated and treated pellets, more remarkable within IFPSCs pellets. Histological analysis confirmed that non-treated IFPSCs formed a poor extracellular matrix (ECM) (Fig. 4b). On the other hand, when ASCs and IFPSCs pellets were cultured with NB260, AB235 or BMP-2 supplementation, cells produced an ECM that was comparable to the native cartilage tissue, although differences between cell sources were found. Haematoxylin-eosin staining of ASCs pellet sections revealed a slightly denser ECM (pink) when compared with IFPSCs pellets sections. Further, glycosaminoglycans (GAGs) staining (blue by alcian blue and purple by toluidine blue) showed a more compact distribution within the ECM produced by ASCs pellet than IFPSCs pellets. Finally, Masson’s trichrome staining revealed an enhanced presence of collagens fibres (green) in ASCs pellet sections when compared with IFPSCs (Fig. 3b and Fig. 4b). Cells embedded in lacunae surrounded by a dense ECM were observed mainly in cell pellets grown in AB235-supplemented cultures, although pellets cultured with NB260 or BMP-2 also presented some lacunae (Fig. 3b and Fig. 4b).

**Fig. 5:**
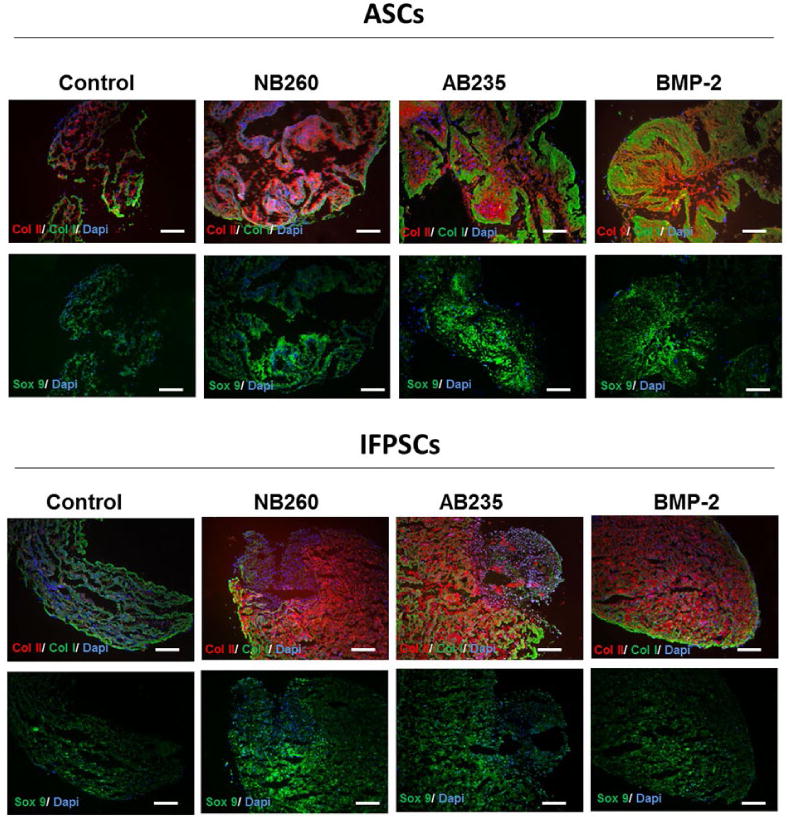
Immunofluorescence staining of pellets sections with ColII (red channel), ColI (green channel) and Sox9 (green channel) antibodies demonstrated an increase of chondrogenic proteins expression and feature spatial distribution induced by the chondrogenic inductors NB260, AB235 and BMP-2. Scale bars: 100 μm.

**Fig. 6:**
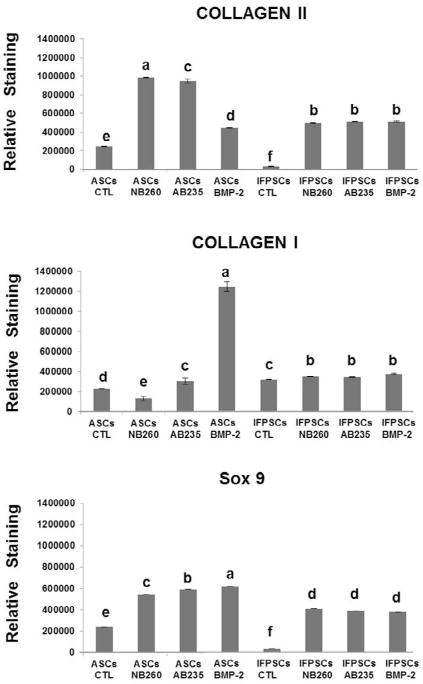
Quantitative image analysis. Immunofluorescence quantification staining in control and NB260-, AB235- or BMP-2-treated pellet sections. Different letters stand for statistically significant differences (one-way ANOVA, *p* < 0.01).

Immunofluorescence analysis for collagen expression showed higher expression of ColII in pellets cultured in NB260- and AB235-containing medium and reduced expression of ColI, restricted to the edges of the pellets. Collagen staining of BMP-2-induced pellets sections showed an expression pattern that was dependent on the cell type: IFPSCs pellets showed a similar expression of ColI and ColII to the pellets induced by AB235 or NB260 addition, whereas ASCs pellets were characterised by an enhanced expression of ColI and a reduced expression of ColII. On the other hand, ASCs control pellets showed lower ColII expression, with a diffuse pattern, when compared with pellets induced by the three growth factors. Interestingly, no expression of ColII was detected in control IFPSCs pellets. Furthermore, pellets cultured in the presence of the growth factors showed enhanced Sox9 expression when compared with control pellets (Fig. 5). Finally, quantitative fluorescence analysis showed a significant ColII and Sox9 increase in intensity (*p* < 0.01) in all factor-induced pellets when compared with control pellets (Fig. 6). Expression of ColI increased significantly (*p* < 0.01) in ASCs pellets treated with BMP-2 *versus* control pellets, while no significant difference in ColI staining was observed in IFPSCs cultured with any of the chondrogenic factors (Fig. 6). Furthermore, NB260 and AB235 supplementation resulted in a significant higher expression (*p* < 0.01) of ColII and Sox9 in ASCs pellets with respect to IFPSCs pellets, while BMP-2 treatment resulted in the same ColII expression in both cell types (Fig. 6). Comparing the effect of each treatment, ASCs pellets induced with NB260 and AB235 showed a significant increase in ColII in contrast to the pellet induced with BMP-2, in which a significant increase in the hypertrophic marker ColI (*p* < 0.01) was observed. In addition, small differences in the staining of Sox9 were observed in ASCs factor-induced pellets. On the other hand, IFPSCs pellets induced with the three different chondrogenic factors showed no significant differences in ColII, ColI and Sox9 fluorescence staining (Fig. 6).

### RT-qPCR analysis

**Fig. 7:**
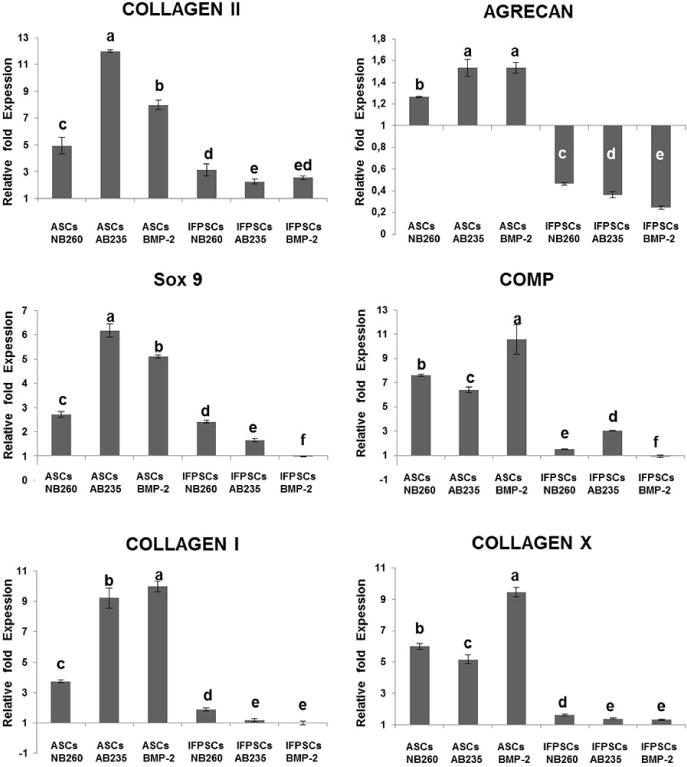
Chondrogenic markers gene expression. RT-qPCR analysis of selected chondrogenic markers after 6 weeks of NB260, AB235 and BMP-2 treatment. All gene expressions were normalised to the untreated control. Different letters stand for statistically significant differences (one-way ANOVA, *p* < 0.01).

RT-qPCR analysis was performed to quantify the expression of genes related to chondrogenic differentiation in ASCs- and IFPSCs-treated and control pellets. In concordance with the histological and immunofluorescence results, ASCs pellets showed a significant (*p* < 0.01) higher expression of *ColII*, aggrecan (*ACAN)*, *Sox9*, cartilage oligomeric matrix protein (*COMP*), *ColI* and *ColX* when compared with IFPSCs pellets (Fig. 7).

When comparing the chondrogenic potential of the tested growth factors, AB235 induced an enhanced expression of the early chondrogenic markers *ColII* and *Sox9* in ASCs-treated pellets. Furthermore, the expression of *ColI* and *ColX*, markers of hypertrophy, was lower in AB235-induced pellets than in BMP-2-induced pellets. Also, a significant lower expression of *ColI* and *ColX* was observed in IFPSCs pellets after AB235 treatment as compared to NB260, while the expression of *ColII* and *Sox9* was increased in NB260-induced IFPSCs pellets when compared to AB235 or BMP-2 supplementation. RT-qPCR analysis showed that ASCs pellets treated with any of the three chondrogenic factors, displayed a higher chondrogenic potential compared to IFPSCs pellets.

### Adipose-derived stem cells treated with AB235 promoted cartilage integration upon transplantation in mice

**Fig. 8:**
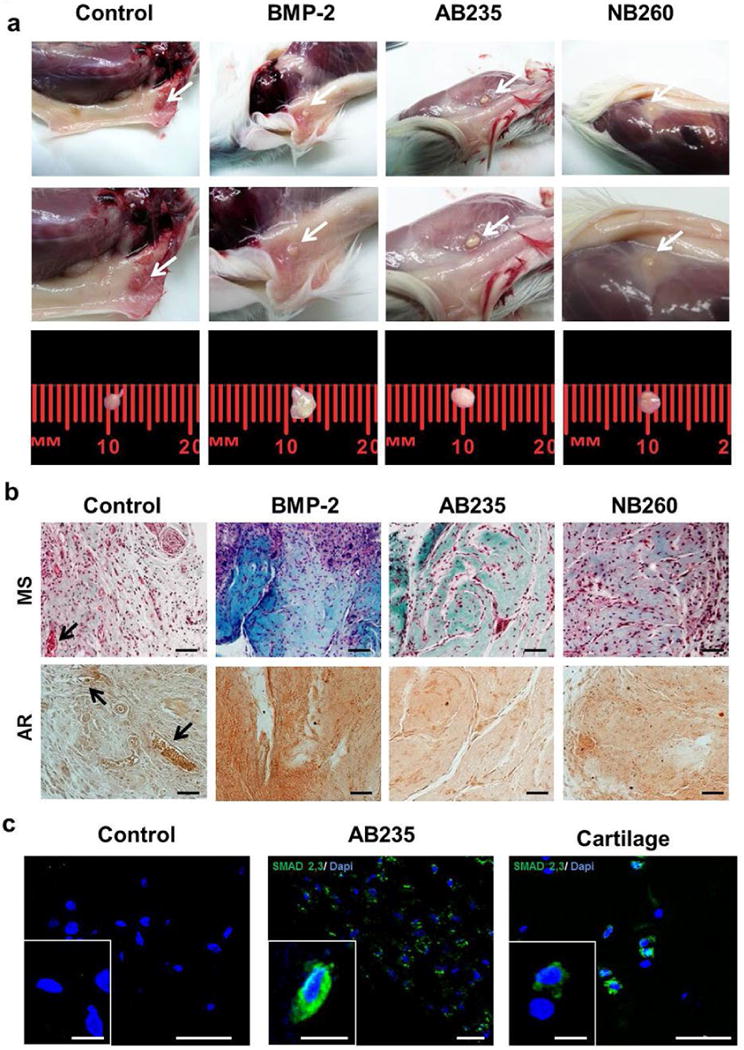
*In vivo* study: subcutaneously transplantation of chondrogenic-induced ASCs pellets. (**a**) White arrows indicate the pellets within the surrounding tissue. (**b**) Harvested pellet sections stained with Masson’s trichrome and alizarin red. Black arrows indicate vascular vessels. Scale bars: 100 μm. p-Smad 2,3 immunostaining of ASCs cultured with and without AB235, cartilage was used as a control. (**c**) Insets show higher magnification of the cells. Scale bars: 50 μm; insert: 10 μm.

An *in vivo* assay was performed to determine if the cartilage-like tissue, growth after chondrogenic induction of ASCs pellets, could integrate into immunodeficient mice. Pellets were subcutaneously transplanted into SCID mice and the stability, integration and vascular invasion of the new cartilage were investigated. After 4 weeks of *in vivo* implantation, all ASCs pellets, previously cultured with AB235 or NB260 chimeric ligands, were recovered (Fig. 8a). Not all BMP-2-treated pellets or control pellets could be recovered because some of them were absorbed by the surrounding tissue (Table 3). Masson’s trichrome staining revealed a robust and dense ECM in the treated pellets, with a significant increment in collagen and GAG production. The largest amount of collagen was found in AB235-and BMP-2-induced pellets, while lack of collagen positive staining was evident in control pellets, which also produced a smaller amount of ECM (Fig. 8b). Alizarin red staining showed strong calcium deposition in pellets cultured with BMP-2 (Fig. 5b), indicating that BMP-2 induced ECM calcification (Fig. 8b). Moreover, the presence of vessels in control pellets was observed (Fig. 8b).

### SMAD2/3 signalling

As TGF-β/SMAD signalling is relevant for cartilage development and maintenance (Wang *et al*., 2014), the expression of phosphorylated-Smad2/3 (p-Smad2/3) protein was analysed by immunofluorescence staining (Fig. 8c). p-Smad2/3 was detectable after 6 weeks of differentiation in AB235-treated ASCs pellets in a comparable pattern to native cartilage tissue. However, no p-Smad2/3 signalling up-regulation was detected in untreated cell pellets (Fig. 8c).

## Discussion

Traumatologists are demanding an effective strategy for a cell-based therapy to treat cartilage lesions and/or tissue degeneration. This comparative study aimed at determining the most suitable combination between two sources of MSCs and three different chondrogenic factors.

ASCs and IFPSCs were cultured under identical conditions and used at the same passage. Furthermore, IFPSCs and ASCs fulfilled the minimal criteria established by the ISCT for defining multipotent mesenchymal stromal cells in terms of adherence to plastic, cell surface marker profile and multidifferentiation capacity (Dominici *et al*., 2006).

For chondrogenic differentiation, both cell types were cultured in the presence of NB260 or AB235 chimeric ligands and BMP-2, in a 3D pellet system that induces cartilage formation (Barna and Niswander, 2007).

Histology and immunostaining confirmed an increment in GAG deposition, an increase in collagen fibres and a higher expression of chondrogenic markers in IFPSCs and ASCs pellets cultured with AB235, NB260 and BMP-2 supplementation, in comparison to control pellets that were smaller and lacking a recognisable tissue organisation. Those findings indicated that a cartilage-like tissue could be obtained from both cellular types when chondrogenic growth factors were employed. However, the characteristics of the new tissue depended on the cell source. RT-qPCR analysis showed a significantly higher expression of the chondrocyte marker genes, *ColII*, *Sox9, ACAN* and *COMP*, in ASCs pellets as compared with IFPSCs. Collagen fibres (ColII) and aggrecan (ACAN) are the predominant component of cartilage ECM (Dell’Accio *et al*., 2001), while COMP is an additional protein present in the cartilage (Hedbom *et al*., 1992). The transcription factor Sox9 is a key marker for early chondrogenesis and acts as a negative regulator of cartilage vascularisation, endochondral ossification and osteogenic differentiation (Liao *et al*., 2014). The most pronounced chondrogenic commitment was found in AB235-treated ASCs when compared to NB260 and BMP-2 treatement, as revealed by the significantly higher transcriptional expression of *CollII* and *Sox9* and the low expression of the hypertrophic markers *ColI* and *ColX* (Vinardell *et al*., 2012). Interestingly, BMP-2 induced higher expression of *ColI* than *ColII* in ASCs pellets. Although increased expression of *ColI* is closely related to *in vitro* chondrogenic differentiation (Tallheden *et al*., 2004), higher expression of *ColI* during chondrogenic differentiation is also associated with the development of a fibro-cartilaginous phenotype (Vinardell *et al*., 2012).

The observed high chondrogenic potential of AB235, in comparison with NB260, may be due to the intrinsic characteristics of the activin/BMP-2 chimeric ligand. Although nodal shares the same signalling pathway with activin, nodal signalling requires the co-receptor cripto to initiate the Smad pathways (Perán *et al*., 2013; Esquivies *et al*., 2014; Kelber *et al*., 2008). Furthermore, activin A is an inhibitor of the catabolic process that leads to the degradation of the ECM (Chang *et al*., 2007; Alexander *et al*., 2007). BMP-2 regulation is a key factor for chondrogenic differentiation and cartilage formation, but high concentrations of BMPs could induce osteogenic differentiation (De Luca *et al*., 2001; Valcourt *et al*., 2002). Thus, the choice of BMP-2 as a chondrogenic factor could be controversial, since its use to induce chondrogenesis has produced contradictory results (Kwon *et al*., 2013; Weiss *et al*., 2010).

After *in vivo* implantation of ASCs pellets, the ECM of pellets cultured with AB235 and BMP-2 supplementation presented greater complexity and denser and richer collagenous fibrils content than NB260-induced pellets or control pellets. Furthermore, AB235-induced pellets showed a viable integration into the surrounding tissue and the capacity to retain a cartilage phenotype in a stable manner, as previously shown (Sekiya *et al*., 2005; Toh *et al*., 2005). In contrast, BMP-2-induced pellets became ossified after being implanted subcutaneously. Resistance to vascularisation and calcification, even at ectopic sites, is essential to achieve functional suitability and stable cartilage (Dell’Accio *et al*., 2001). Therefore, the calcification of BMP-2 treated pellets after ectopic transplantation in immune-deficient mice could indicate that BMP-2 media supplementation is not a suitable approach for cell-based therapy of articular cartilage (Pelttari *et al*., 2008; Dickhut *et al*., 2009).

Previous studies show that MSCs, including that from joint tissues, fail to form stable cartilaginous tissue *in vivo* after subcutaneous implantation (Vinardell *et al*., 2012; De Bari *et al*., 2001; Marsano *et al*., 2007). These studies highlight the need for novel approaches to promote a more stable chondrogenic phenotype by using environmental cues, encapsulation in an appropriate biomaterial or new signalling molecules, among others. In this study, it was shown that stimulation of chondrogenesis with the chimeric ligand AB235 formed a stable *in vitro* and *in vivo* cartilaginous-like tissue.

TGF-β/Smad signalling is relevant in cartilage development and maintenance. While TGF-β subfamily ligands (TGF-β, activins, nodals, myostatin and Mullerian-inhibiting substance) activate Smad2/3 transcription factors, Smad1/5/8 transcription factors are activated by the BMP pathway (Wang *et al*., 2014). Both, Smad2/3 and Smad1/5/8 phosphorylation are required for the onset of chondrogenic differentiation; however, during later stages of chondrogenesis, Smad2/3 phosphorylation participates in ColII deposition and blocks terminal differentiation, while Smad1/5/8 phosphorylation modulates chondrogenic hypertrophy (Hellingman *et al*., 2011). An enhanced expression of p-Smad2/3, after induction with AB235, was observed when compared with control cells. As AB235 chimeric ligand has the signalling properties of activin, the involvement of Smad2/3 pathway in the chondrogenic stimulation of the pellets in response to AB235 chimeric ligands may contribute to the maintenance of the cartilage phenotype (Diederichs *et al*., 2016).

With the aim of optimising chondrogenic induction protocols, two different cell sources were evaluated. The observed higher chondrogenic potential of ASCs in comparison with IFPSCs could be attributed to different factors, such as the niche characteristics or cell proliferation rate. According to the micro-environmental niche theory, IFPSCs should have higher chondrogenic potential than ASCs due to their proximity to the cartilage (Liu *et al*., 2014; O’Sullivan *et al*., 2011). Nevertheless, IFP cells from patients with OA have an inflammatory phenotype, which may have lasting effects on the differentiation ability of stem cells isolated from this tissue (Klein-Wieringa *et al*., 2011). Other studies suggest that stem cells from patients with advanced OA may display a diminished chondrogenic capacity (Murphy *et al*., 2002; Diekman and Guilak, 2013). Conversely, IFPSCs isolated from OA donors possess a comparable chondrogenic capacity to IFPSCs isolated from patients undergoing ligament reconstruction (Liu *et al*., 2014). It is proposed that faster growing cells show a superior chondrogenic differentiation, suggesting a positive correlation between proliferation rate before start of high-density culture and chondrogenesis (Dexheimer *et al*., 2012). In the current study, ASC and IFP cells presented similar, although not identical, doubling times, suggesting that these slight differences in proliferation rates could play a discreet role in the higher chondrogenic potential of ASCs.

## Conclusions

This study demonstrated that MSCs isolated from liposuctions had higher chondrogenic potential than those isolated from the IFP of OA patients. An efficient and reproducible protocol for chondrogenic differentiation – based on the use of AB235 chimeric ligand – with a therapeutic potential in patients with OA, was presented.

## Discussion with Reviewers

Reviewer 1 Most investigators use TGF-β as the prototypic inducer of chondrogenesis. Why did you choose instead to use BMP-2, which promotes the formation of hypertrophic chondrocytes and bone?

Authors BMPs are members of the TGF-β protein superfamily, whose members can induce chondrogenesis Kolf *et al*., 2007, additional reference). BMP-2 was used because it can induce chondrogenesis with characteristic similar to other factors belonging to the TGF-β family (Rocha *et al*., 2012; Liao *et al*., 2014, additional references).

Reviewer 1 Have you any data to suggest that your heterodimers are less hypertrophic-inducing than TGF-β?

Authors TGF-β was not used in the current work to induce chondrogenesis. Induction of chondrogenesis in MSCs with TGF-β leads to a hypertrophic phenotype (Mueller *et al*., 2010, additional reference), which could be avoided when using the chimeric ligand described in the current work.

## Figures and Tables

**Table 1:** OA patients-related information. Patient data and evaluation of the conditions of the knee according the Ahlback scale value and the Knee Society Knee Scoring System (KSS).

Patient	Gender (M/F)	Age (years)	Ahlback	KSS
1	M	66	III	30
2	F	74	III	28
3	M	67	II	32
4	M	74	III	25
5	M	59	III	37
6	F	70	III	29
7	F	68	III	30
8	F	57	II	35

M: male patient; F: female patient.

**Table 2:** Sequences of the primers used for RT-qPCR analysis.

Gene	Forward	Reverse
***ColI***	ATGGATGAGGAAACTGGCAACT	GCCATCGACAAGAACAGTGTAAGT
***ColII***	GAGACAGCATGACGCCGAG	GCGGATGCTCTCAATCTGGT
***ColX***	GCCCACTACCCAACAC	TGGTTTCCCTACAGCTGA
***COMP***	AACACGGTCACGGATGACGACTATG	CACAGAGCGTTCCGCAGCTGTTC
***ACAN***	AGGATGGCTTCCACCAGTGC	TGCGTAAAAGACCTCACCCTCC
***Sox9***	ACTCCGAGACGTGGACATC	TGTAGGTGACCTGGCCGTG
***GAPDH***	TGCACCACCAACTGCTTAGC	GGCATGGACTGTGGTCATGAG

**Table 3:** Number of pellets recovered after 4 weeks of *in vivo* assay.

	CTL	NB260	AB235	BMP2
**ASCs**	2/3	3/3	3/3	1/3
